# Tranexamic Acid Application in Facial Aesthetic Surgery: An Umbrella Review

**DOI:** 10.1093/asjof/ojae105

**Published:** 2024-11-07

**Authors:** Mohamed Badie Ahmed, Deemah Assami, Dima Nasrallah, Fatima Saoud Al-Mohannadi, Salwa Al-Maraghi, Abdelrahman Badie Ahmed, Abeer Alsherawi

## Abstract

Tranexamic acid (TXA) is an antifibrinolytic agent that is considered as one of the latest interventions currently being investigated in the field of facial aesthetic surgeries, as it is predicted to be effective in reducing intraoperative and postoperative complications of facial aesthetic surgeries. This review focuses on giving readers a comprehensive overview regarding the use of TXA in facial aesthetic surgeries. In this umbrella review, data were extracted from existing systematic reviews and meta-analysis that focused on the use of TXA in facial aesthetic surgeries. The authors searched PubMed, Embase, and Scopus databases. The data were extracted using a standard format, and the AMSTAR-2 (A Measurement Tool to Assess Systematic Reviews) tool was used to assess the quality of the included reviews. In total, this study included 14 systematic reviews and meta-analyses all of which evaluated the effect of TXA on facial aesthetic surgeries, which included rhinoplasty, septorhinoplasty, rhytidectomy, and blepharoplasty. The majority of the included studies focused on reporting the effect of TXA on blood loss volume (BLV) and duration of surgery (DOS) as well as other postoperative complications. Eleven out of the 12 studies that focused on rhinoplasty showed that TXA used reduced BLV. In addition, in 8 studies that were focusing on DOS, the majority showed a reduction in DOS with TXA use. While in the case of septorhinoplasty, 3 studies revealed that TXA use decreased BLV. Moreover, in the case of rhytidectomy, 3 out of 6 systematic reviews showed reduction in BLV, while 2 reported reductions in DOS. Finally, the authors conclude that the use of TXA is indeed efficient in reducing BLV and DOS, in addition to some of the complications that can occur during or after facial aesthetic surgeries. However, in order to reach a final decision on the implication of the use of TXA in facial aesthetic surgeries, further studies should be established using a standardized protocol in assessing the desired outcomes.

Tranexamic acid (TXA) is a synthetic lysine-analog antifibrinolytic that is responsible for inhibiting the conversion of plasminogen into plasmin (Figure 1). This is going to inhibit the degradation and disintegration of fibrin clots.^1^ Nevertheless, the use of TXA can reduce bleeding and inflammatory reactions which is why it has been currently used in certain surgical procedures, such as orthopedic surgery, trauma surgery, and cardiac surgery.^2,3^ TXA has been shown to have an effective role in minimizing postoperative complications, such as blood loss and blood transfusions, which are commonly encountered in such procedures.^4^ However, it is important to take into consideration that just like any other medication, the use of TXA can have potential side effects on patients, such as thromboembolic events, but in most patients, TXA is well tolerated. In addition, it is important to highlight that at high doses, TXA may cause significant side effects, such as seizures.^5^ Therefore, this medication should be administered when there is a proper indication, and at the correct dose and frequency.

**Figure 1.**
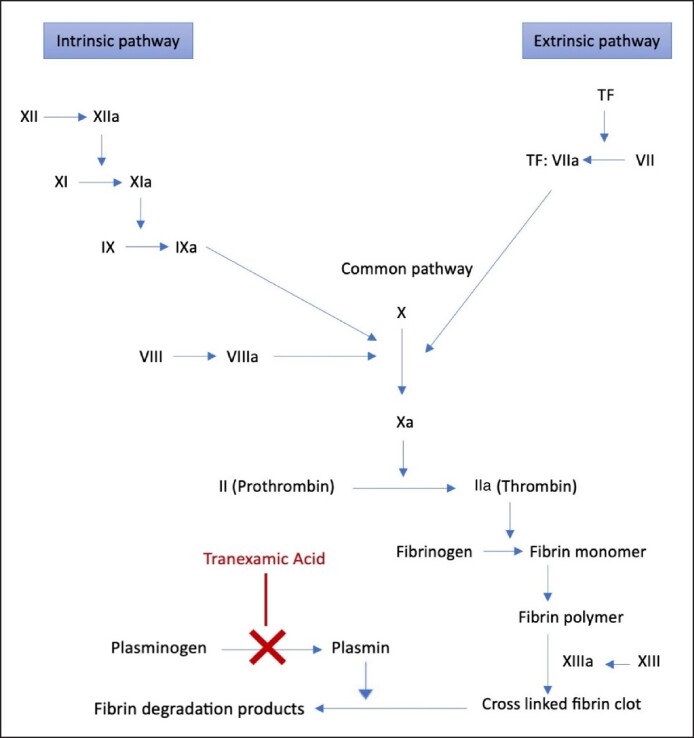
The coagulation cascade and the mechanism of action of tranexamic acid.

The number of aesthetic procedures performed has risen dramatically in recent years. Hemostasis in aesthetic surgeries is considered one of the most important aspects that should be looked after because blood loss can complicate such surgeries and increase their morbidity and mortality.^6^ Hence, various studies were conducted to assess the use of multiple different interventions, aiming to reduce the severity of ecchymosis, bruising, and edema postoperatively, in facial aesthetic surgeries, such as rhinoplasty, blepharoplasty, rhytidectomy, and neck lift. However, one of the most recent interventions that is being investigated in the field of aesthetic surgery is TXA, which plays a crucial role in the stabilization of the fibrin meshwork.^7^ Hence, reducing complications, such as hematomas and ecchymosis, can lead to cutaneous vascular compromise, as well as permanent pigmentation changes.^8^

All these complications are associated with longer recovery time and hospital stay as well as poorer outcome and patient satisfaction. Moreover, multiple clinical trials were conducted to assess the use of different medications, surgical methods, and other interventions to prevent postoperative complications or at least to minimize them.^9^ Therefore, with the current knowledge of the mechanism of action of TXA, it is currently being investigated as an intervention to prevent complications in facial aesthetic surgeries.^10^ As an example, recent studies revealed that the use of TXA has been shown to be effective in reducing intraoperative bleeding as well as postoperative edema and ecchymosis following rhinoplasty. And so, there is an anticipation that the use of TXA can play an important role in reducing postoperative complications of facial aesthetic surgeries.^11^

In this study, we performed an umbrella review to examine the evidence synthesis, both systematic reviews (SR) and meta-analysis (MA), and summarize their findings on papers focusing on the application of TXA in aesthetic facial surgeries and its efficacy in limiting the complications of facial aesthetic surgeries both intraoperatively and postoperatively. In addition, we aim to provide a clear overview of a wide topic field to clinicians and to identify future implications of TXA in the field of facial aesthetic surgery. Hence, our umbrella review aims to provide readers with an update on current understanding and clinical suggestions about TXA's effectiveness in facial plastic surgery that are supported by high-caliber research.

## METHODS

### Search Strategy and Study Selection

We performed an umbrella review, which is a systematic collection and evaluation of multiple systematic reviews and meta-analyses.^12^ The guidelines that were followed in this review were the PRISMA guidelines. The search for evidence synthesized data on the use of TXA in facial aesthetic surgery and its effects was conducted on June 5, 2024. The search terms that were used to identify potential articles were as follows: TXA, facial plastic surgery, facial aesthetic surgery, blepharoplasty, septorhinoplasty, platysmaplasty, neck lift, rhytidectomy, or facelift. We searched the PubMed, Embase, and Scopus databases without any restriction of date or publication. However, the exclusion criteria were the non-English and animal studies, as well as craniofacial and other reconstructive, postbariatric, and other aesthetic plastic surgeries. The results were uploaded on Rayyan software for screening.^13^ Two independent reviewers screened titles and abstracts to assess eligibility for inclusion, followed by full-text screening. Any conflict was resolved by a third reviewer, followed by discussion and agreement between the reviewers.

The search strings used are shown in Supplemental Figure S1. Furthermore, reference lists of included papers were screened for additional relevant reviews that might have been missed during the initial search. The extraction of data was done regarding the synthesis type (SR or MA), title and author, year of publication, the use of intraoperative TXA administration in facial aesthetic surgery (route and dosage), a summary of included studies, follow-up postoperatively, and possible evidence gaps. The primary findings were summarized concerning the reduction of intraoperative bleeding and surgery related outcomes (DOS, edema, ecchymosis, etc.).

### Quality Assessment

The AMSTAR-2 (A Measurement Tool to Assess Systematic Reviews) was utilized to evaluate the quality of each included study in this umbrella review. AMSTAR-2 assesses each study based on 16 different safeguards to determine its methodological rigor and reliability.

### Data Synthesis

A structured summary of findings was done for the eligible and included SRs and MAs. The effects of the use of TXA in facial aesthetic surgery were assessed in 4 categories: rhinoplasty, septorhinoplasty, rhytidectomy, and blepharoplasty. A separate table of findings was formulated for each of the categories.

## RESULTS

### Characteristics of Included Studies

**Figure 2.**
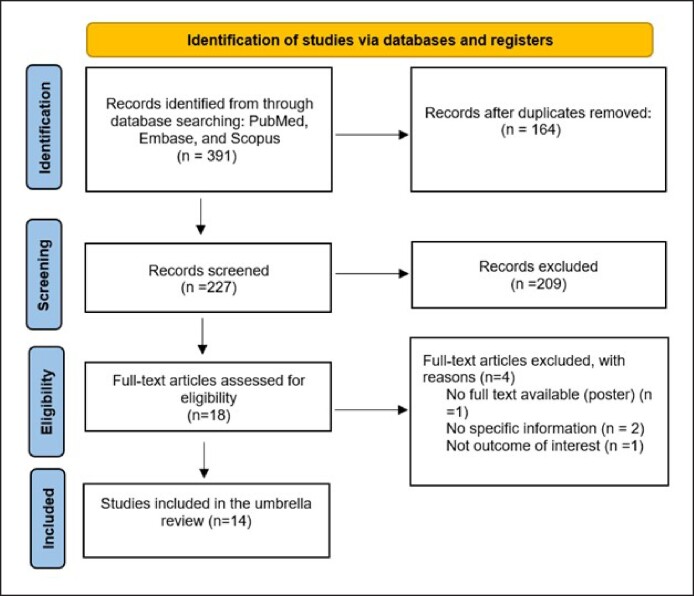
PRISMA flow diagram for the included studies.

The primary systematic database search in PubMed, Embase, and Scopus identified a total of 391 records (Figure 2). After removing duplicates, a total of 227 records underwent title/abstract screening, resulting in 18 eligible records for full-text evaluation based on the inclusion/exclusion criteria. Of these 18 meta-analyses and/or systematic reviews, a total of 4 reviews were excluded for the following reasons: lack of full text (poster; *n* = 1), lack of specific information (*n* = 2), and absence of outcome of interest (*n* = 1). Table 1 summarizes the characteristics of the included studies, which encompass 4 main types of plastic surgery: rhinoplasty, septorhinoplasty, rhytidectomy, and blepharoplasty. All included reviews were published in the period spanning from 2018 to 2024. A total of 14 reviews were included in this umbrella review, each examining one or more of the procedures mentioned: 12 for rhinoplasty, 3 for septorhinoplasty, 6 for rhytidectomy, and 5 for blepharoplasty.

**Table 1.** Syntheses that Include Rhinoplasty

	Synthesis author and year	Synthesis type	No. of articles included	Aims		Route	Dosage	No. of participants included
1	Goncalves et al (2023)	SR/MA	6	BLV	Reduced significantly (SMD 0.69, *P* = .02)	IV (5)/oral (1)	10-1000 mg/kg	366
				DOS	Statistically nonsignificant reduction (SMD 0.26, *P* = .09)			
				Thromboembolic events	No events			
2	Wang et al (2024)	SR/MA	5	BLV	Statistically nonsignificant reduction (MD = −0.53; 95% CI [−1.15 to 0.09])	IV	Not reported	326
				DOS	No significant difference (SMD −0.03; CI 95% [−0.32 to 0.25])			
				Surgical field	Statistically significantly improved			
3	Gutierrez et al (2024)	SR/MA	11	BLV	Reduced significantly (MD = −39.37 mL; 95% CI [−62.70 to −16.05 mL]; *P* = .0009)	IV (6)/oral (2)/topical (2)/local injection (1)	5 mg/kg-10 mg/kg-1 g	841
				Postoperative edema	Decreased significantly (MD = −0.78; 95% CI [−1.28 to −0.27 points]; *P* = .003)			
				Postoperative ecchymosis	Decreased significantly POD1 (MD = −1.13; 95% CI [−1.99 to −0.28]; *P* = .01)			
				DOS	No difference (SMD = −0.26; 95% CI [−0.56 to 0.04]; *P* = .09)			
				Surgeon satisfaction	Increased significantly (SMD = 1.55; 95% CI [0.33 to 2.77]; *P* = .01)			
4	Laikhter et al (2022)	SR/MA	6	BLV	Reduced significantly (MD = −26.3 mL; 95% CI [−40.0 to −12.7 mL]; *P* < .001)	IV (4)/oral (2)	Not reported	Not reported
				Postoperative edema	Decreased significantly			
				Postoperative ecchymosis	Decreased significantly (*P* < .05)			
				Surgeon satisfaction	Increased significantly			
				Postoperative hemoglobin	No statistical Significance			
				Postoperative hematocrit	Reduced significantly			
5	AlGhanim et al (2021)	SR	4	BLV	Reduced significantly	IV (3)/oral (1)	10 mg/kg-1 g	Not reported
				Postoperative edema	Decreased significantly			
				Postoperative ecchymosis	Decreased significantly			
				DOS	Reduced significantly			
				Surgical field	Improved significantly			
6	Locketz et al (2020)	SR	6	BLV	Reduced significantly	IV (3)/oral (3)	10 mg/kg-500 mg-1 g	384
				Postoperative edema	Reduced significantly			
				Postoperative ecchymosis	Reduced significantly			
				DOS	Shorter duration			
7	McGuire et al (2019)	SR/MA	5	BLV	Reduced significantly (MD = −41.6 mL; 95% CI [−69.8 to −13.4 mL]	IV (3)/oral (2)	10 mg/kg-1 g	332
				Postoperative edema	Reduced significantly			
				Postoperative ecchymosis	Reduced significantly			
8	Ping et al (2019)	SR/MA	3	BLV	Reduced significantly (MD = −46.8, 95% CI, [−70.64 to −22.96], *P* < .001)	IV (2)/oral (1)	10 mg/kg-1 g	130
				Postoperative edema	Reduced significantly			
				Postoperative ecchymosis	Reduced significantly			
9	De Vasconcellos et al (2018)	SR/MA	5	BLV	Reduced significantly (MD = −42.28 mL, 95% CI [−70.36 to −14.21 mL])	IV (3)/oral (2)	10 mg/kg-1 g	276
				Postoperative edema	Reduced significantly			
				Postoperative ecchymosis	Reduced significantly			
				Thromboembolic events	No events			
10	Siotou et al (2019)	SR/MA	3	BLV	Reduced significantly (MD, −32.48 mL; 95% CI [−56.98 to −7.98 mL]; *P* = .009)	IV (2)/oral (1)	10 mL/kg-1 g	196
				DOS	No significant difference			
11	Scarafoni (2021)	SR	6	BLV	Reduced significantly	IV (3)/oral (1)/both (2)	10 mg/kg-1 g	532
				Postoperative edema	Decreased			
				Postoperative ecchymosis	Decreased			
				Thromboembolic events	No events			
12	Yap et al (2021)	MA	3	BLV	Reduced significantly (SMD, −55.05; 95% CI [−72.45 to −37.66])	IV (1)/oral (2)	10 mg/kg-1 g	155
				Surgical field	Worsens (SMD, 1.60; 95% CI 1.32-1.88)			
				DOS	Reduced significantly −0.39 (95% CI −0.69 to −0.09)			

BLV, blood loss volume; DOS, duration of surgery; IV, intravenous; MA, meta-analysis; SMD, standard mean difference; SR, systematic reviews; TXA, tranexamic acid.

**Figure 3.**
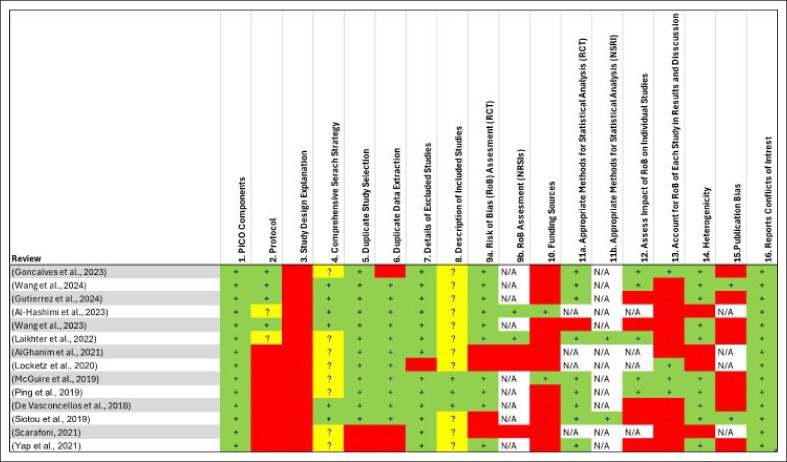
Quality assessment of the 14 syntheses included.

AMSTAR-2 outcomes showed that all included studies exhibited the PICO tool components to formulate their research question, which are patients/population, intervention, control, outcome, and the reports of conflict-of-interest domains. On the other hand, none reported study design explanation domain, and little reported the funding sources of each paper included in the different meta-analysis. The various safeguards against each included study are shown in Figure 3.

### Rhinoplasty

Of the included reviews, 12 SRs and/or MAs looked into TXA in rhinoplasty (Table 1). In those reviews, 11 out of the 12 reviews reported the effect of TXA on blood loss volume (BLV), making it the most commonly investigated outcome. The second most frequently researched outcome is the DOS and postoperative ecchymosis and edema, with 7 reviews reporting it. This is followed by thromboembolic events that were examined in 3 reviews. Other effects studied were surgical field quality, surgeon satisfaction, postoperative hemoglobin and hematocrit, and transfusion occurrence.

#### Blood Loss Volume

Among the 12 reviews examining TXA's effect on BLV, 11 of the reviews showed results that aligned, indicating that TXA significantly decreased BLV in rhinoplasty procedures.^14-16^ Even so, the degree of reduction in blood loss varied among different studies, as shown in Table 1. However, Wang et al had findings that were conflicting with the literature as they reported a statistically insignificant association between BLV and the use of TXA.^10^ Furthermore, de Vasconcellos et al examined whether the route of TXA administration would affect the degree of reduction in BLV. They found that both routes showed statistical significance in reduction of BLV with the oral route being more effective.^11^

#### Postoperative Ecchymosis and Edema

In regards to the role of TXA in postoperative ecchymosis and edema, 8 reviews consistently demonstrated a statistically significant reduction in both postoperative ecchymosis and edema.^3,11,16-21^ Gutierrez et al concluded that TXA reduced the degree of edema and ecchymosis on postoperative Day 1.^17^ Additionally, de Vasconcellos et al found, on the first postoperative week, that the TXA group had significantly lower scores of lower eyelid edema.^11^

#### Duration of Surgery

Seven reviews examined the difference in DOS when TXA was used.^3,10,15,17,20,22^ Three of which found that TXA reduced DOS; however, 4 papers found that the reduction was not statistically significant.

#### Other Outcomes Explored

Another outcome explored in 3 reviews was the occurrence of thromboembolic events when TXA is used in rhinoplasty. All 3 unanimously reported that no thromboembolic event occurred.^11,14,21^ Furthermore, surgical field assessment as an outcome of using TXA was investigated in 3 reviews, in which 2 found TXA improved the surgical field, whereas 1 found it worsened it.^10,15,16^ Surgeon satisfaction as a result of TXA use was another outcome of interest in 2 reviews, both of which found that TXA effectively improved surgeon satisfaction.^17,18^ Lastly, postoperative hemoglobin and hematocrit were investigated by Laikhter et al. Although there was a significant difference between preoperative and postoperative hematocrit levels, the change in hemoglobin was insignificant.^18^

### Septorhinoplasty

Three reviews included in this umbrella review compared different effects of TXA in septorhinoplasty procedures, as shown in Table 2.^20,21,23^ One of the outcomes studied was the relationship between TXA and blood loss, with all 3 identifying a significant reduction in BLV. Another outcome explored by 2 reviews was the effect of TXA on operation time, and the results affirmed that there was no significant difference when TXA was used.^20,23^ Moreover, TXA was found to significantly improve postoperative edema and ecchymosis scores when compared with control groups in 2 of the included reviews.^20,21^ Elena Scarafoni found a significant association between the use of TXA, BLV, postoperative edema, and postoperative ecchymosis compared with the placebo group. However, when the use of TXA was compared with the use of corticosteroid, there was no statistical significance.

**Table 2.** Syntheses that Include Septorhinoplasty

	Synthesis author and year	Synthesis type	No. of articles included	Aims		Route	Dose	No. of participants included
1	Wang et al (2023)	SR/MA	2	BLV	Significant reduction (SMD −1.05, 95% CI [−1.72 to −0.38])	Local	Not reported	258
				DOS	Significant reduction (SMD −3.09, 95% CI [−3.85 to −2.33])			
				Surgical field	Improved significantly			
2	Ping et al (2019)	SR/MA	1	BLV	Significant reduction (SMD −45, 95% CI [−71.03 to −18.97])	Oral	1 g	75
				Postoperative edema	Significantly improved			
				Postoperative ecchymosis	Significantly improved			
3	Scarafoni (2021)	SR	1	BLV	Significant reduction	Oral	1 g	75
				Postoperative edema	Significantly improved			
				Postoperative ecchymosis	Significantly improved			

BLV, blood loss volume; DOS, duration of surgery; MA, meta-analysis; SMD, standard mean difference; SR, systematic reviews; TXA, tranexamic acid.

### Rhytidectomy

A total of 6 SRs examined the effect of using TXA in rhytidectomy, with only one of these SRs conducting a meta-analysis (Table 3). The most commonly reported endpoints were postoperative edema and ecchymosis as well as postoperative drainage, in which 5 reviews examined this relationship. BLV and DOS were the second most reported outcomes with 3 reviews investigating them. Other outcomes examined were hematoma rate and thromboembolic events.

**Table 3.** Syntheses that Include Rhytidectomy

	Synthesis author and year	Synthesis type	No. of articles included	Aims		Dose and route	No. of participants included
1	Scarafoni et al (2021)	SR	5	BLV	Majority had significant reduction	Soaked pledgets under the skin flap (1) 1 g IV before and 4 h after surgery (1) Either 1 or 9.1 mg/mL subcutaneously (3)	243
				Postoperative drainage output	Significant reduction		
				Postoperative edema and ecchymosis	Significant reduction		
				Thromboembolic events	1 case reported, not statistically significant		
				DOS	1 study: 25 to 60 min reduction		
2	Locketz et al (2020)	SR	4	BLV	1 study: significant reduction/1 study: insignificant reduction	Soaked pledgets under the skin flap (1) 1 g IV before and 4 h after surgery (1) Either 1 mg/mL or 9.1 mg/ mL Subcutaneously (2)	204
				Postoperative drainage output	Significant reduction		
				Postoperative edema and ecchymosis	Significant reduction		
				Thromboembolic events	No events		
				DOS	1 study: 25 to 60 min reduction		
3	Laikhter et al (2022)	SR and MA	3	Postoperative drainage output	Significant reduction	Not specified	150
				Postoperative edema and ecchymosis	Significant reduction		
				Hematoma rate	Insignificant difference in rate		
4	Al-Hashimi et al (2023)	SR	3	Postoperative drainage output	Significant reduction	1 g IV before and 4 h after surgery (1) Irrigation with 20 mL of 2.5% TXA solution (1) 9.1 mg of TXA/mL subcutaneously (1)	150
				Postoperative edema and ecchymosis	Significant reduction		
				Hematoma rate	Insignificant difference in rate		
5	Wang et al (2024)	SR and MA	1	DOS	TXA: minimum operation time = 270 min (range, 172-401 min)	1 g IV before and 4 h after surgery	44
					Controls: minimum = 256 min (range, 205-330 min)		
					Significance was not reported		
6	AlGhanim et al (2021)	SR	4	BLV	Improved	Soaked pledgets under the skin flap (1) 1 g IV before and 4 h after surgery (1) Either 1 or 9.1 mg/mL subcutaneously (2)	Not reported
				Postoperative drainage output	Significant reduction		
				Postoperative edema and ecchymosis	Significant reduction		

BLV, blood loss volume; DOS, duration of surgery; IV, intravenous; MA, meta-analysis; SR, systematic reviews; TXA, tranexamic acid.

#### Blood Loss Volume and Drainage Output

Half of the included SR focusing on the association between TXA and rhytidectomy assessed the change in BLV.^3,21,24^ All 3 reviews consistently demonstrated a reduction in BLV among patients who received TXA compared with those given a placebo. While most studies included reported that this reduction is significant, a randomized controlled trial included in all the 3 SRs found that this reduction in BLV was not significant compared with placebo.^3^ However, Elena Scarafoni concluded that the route of TXA administration, whether oral, intravenous (IV), or local injection, does not significantly impact BLV. This suggests that BLV might not be a determining factor when choosing the route of administration for TXA, as all methods resulted in comparable reductions in BLV.^21^ Furthermore, 5 of the included reviews delved into the difference in drainage output between TXA group and controls.^3,16,18,21,24^ All their findings were aligning, indicating a significant reduction in drainage output in TXA group by around 70%. Additionally, Locketz et al reported that 77.3% of the TXA group patients removed the surgical drain only 1 day after the operation, compared with only 34.4% of the control group (*P* < .001).^3^

#### Duration of Surgery and Time to Achieve Homeostasis

DOS was examined in 3 of the included reviews.^3,10,21^ Wang et al excluded the study from the analysis because of the heterogeneity of its results, as they were unable to draw a definitive conclusion.^10^ Locketz et al and Elena Scarafoni concluded that there was approximately a 25 to 60 min reduction in DOS per patient in TXA group compared with placebo group.^3,21^ It was also noted that the time to achieve homeostasis was 12.9 min (±4.2) compared with 20 to 30 min in the control group. However, no inference about the statistical or clinical significance of these differences was reported.

#### Postoperative Ecchymosis, Edema, Hematoma, and Side Effects

Five out of the 6 reviews reviewed the complications and side effects associated with the use of TXA in rhytidectomy.^3,16,18,21,24^ The complications investigated included postoperative edema, ecchymosis, and hematoma, while the side effects were generally not specified but occasionally included thromboembolic events. There was heterogeneity in the system used to report ecchymosis and edema; some relied solely on patients’ ratings, others on surgeons’ assessment, and some on both. Nevertheless, none of the reviews employed objective assessment measures. Generally, there was a trend of decreased postoperative ecchymosis and edema in patients who received TXA compared with controls, whether it was patient rated or surgeon assessed. However, the only statistically significant difference was in the surgeon-rated ecchymosis.^3,18,24^ In regard to postoperative hematoma, the findings were inconsistent. While some reviews established no difference in hematoma between intervention and control groups,^21^ other reviews inferred a lower incidence in the TXA group, though this difference was not significant.^16,18,24^ As for the side effects, none of the included reviews ascertained any adverse effects, except for 1 nonstatistically significant thromboembolic event in the TXA group, which was locally injected, noted by Elena Scarafoni.^21^

### Blepharoplasty

Among the included reviews, a total of 5 SR and/or MA investigated the effect of utilizing TXA in blepharoplasty in terms of BLV, postoperative complications including ecchymosis and edema, and DOS in all of the reviews, as illustrated in Table 4. As for BLV, it was concluded that there was no difference between the TXA treatment group and the control group.^3,16,18,21,23^ Nonetheless, in a meta-analysis that included blepharoplasty trial, among other reviews, it was concluded that there is a significant reduction in BLV with standard mean difference (SMD) of −1.05 (95% CI −1.72 to −0.38).^23^ Similarly, when comparing postoperative edema and ecchymosis between the intervention and control group, it was found that there were no differences between them.^16^ Additionally, there was no effect of using TXA on DOS in blepharoplasty, which was also supported by the meta-analysis conducted by Wang et al.^3,18,21,23^ Finally, an analysis was conducted to determine whether TXA has pain-attenuation effect; however, this difference was also found to be insignificant.^16,21^

**Table 4.** Syntheses that Include Blepharoplasty

	Synthesis author and year	Synthesis type	No. of articles included	BLV	Postoperative complications	Duration of surgery (DOS)	Dose and route	No. of participants included
1	Scarafoni et al (2021)	SR	1 upper eyelid blepharoplasty	No difference in BLV	Insignificant reduction in postoperative ecchymosis	No difference in DOS	2% Lidocaine with 100 mg/mL of TXA subcutaneously injected	34
	Locketz et al (2020)							
	Laikhter et al (2022)							
	Wang et al (2023)							
	AlGhanim et al (2021)							

BLV, blood loss volume; DOS, duration of surgery; TXA, tranexamic acid.

## DISCUSSION

TXA, as an effective agent that minimizes blood loss, has gained enormous value in plastic surgery practice. It facilitates hemostasis during and after surgery, thereby reducing blood loss and the likelihood of transfusion. In plastic surgery procedures where aesthetics is a critical determinant of success, bleeding may obscure fields, complicate the surgery, and deteriorate the quality of the result. Reducing intraoperative and postoperative bleeding, TXA helps to make surgery not only safer but also more comfortable for a patient, avoiding the formation of hematomas, bruises, skin necrosis, and making it easier for a surgeon to operate.

TXA has been researched with regards to the field of plastic surgery, where many clinical trials and reviews have demonstrated the advantages of using the drug.^21^ Based on the literature review, it was agreed and concluded that TXA is a reliable agent in the reduction of blood loss in different types of plastic surgery, including rhinoplasty and rhytidectomy. These reviews demonstrate that regardless of whether TXA was administered topically, intravenously, or orally, it reduces intraoperative blood loss and the development of postoperative hematomas. The most common TXA regimen used is an IV bolus of 10 mg/kg followed by a continuous infusion of 5 mg/kg/h. However, several studies show that both TXA oral and topical forms can be as efficacious as IV TXA. Nonetheless, the half-lives of IV TXA and oral TXA are 2 and 10 h, respectively.^16^

In addition, the reviews included in our umbrella review were assessed using AMSTAR-2 tool. The results in Figure 3 highlighted variability in adherence to multiple quality domains. All the papers adhered to defining PICO components and reporting conflicts of interest, which are considered key quality factors. However, many papers did not adhere to the following domains: comprehensive search strategies, duplicate study selection, and risk of bias assessments. In general, the results reflect variability in adherence to the quality domains, and this suggests that methodological improvements are needed in many papers.

### Blood Loss Volume

Regarding rhinoplasty procedures, TXA has been found to decrease BLV in 11 reviews. In addition, the authors investigated the effect of TXA in rhytidectomy in 6 SRs and reduction of BLV in 3 SRs. In spite of that, in 4 SRs and/or MAs, it was found that TXA had no effect in reducing BLV in patients who underwent blepharoplasty.

Reduction of blood loss is essential for patient safety as it can indirectly reduce the incidence of blood transfusion by aiding in securing hemostasis and decreasing hematoma formation. In addition, it can increase the visibility of the surgical field, making it possible to accomplish precise and efficient movements during operations and improving results, hence increasing surgeons’ satisfaction rate.

Upon reviewing the literature, in almost all studies, the authors investigate the outcome of TXA on BLV which showed that TXA significantly reduced blood loss in facial aesthetic surgery. However, the effect of mode of administration and dosage on blood loss is still yet to be explored. Different SR and/or MA researched variable timings, dosages, and routes of TXA; but there is 1 paper that compared the amount of blood loss after usage of both oral and IV TXA in patients undergoing rhinoplasty procedures. The results favor administering preoperative oral TXA over IV TXA, as oral TXAs have resulted in a greater reduction of blood loss. This could be explained by the different peak plasma concentrations of each TXA drug form. Studies suggest that oral TXA reaches its peak plasma concentrations within 2 to 4 h, while IV TXA concentration levels reach subtherapeutic levels within 6 h.^11^ Another important reported outcome of TXA is the reduction of blood transfusion occurrences. In 1 SR/MA, the effect of IV TXA on patients undergoing both cosmetic and reconstructive surgeries was studied. The IV TXA group had a significantly lower risk of blood transfusion incidence compared with the control group.^10^ Furthermore, several papers examined the quality of surgical fields as well as surgeon's satisfaction rate after the administration of TXA. Most of the studies concluded that TXA resulted in a clearer surgical field and a higher surgeon satisfaction rate. Nonetheless, intraoperative blood loss is subjective to the surgeon. In addition, a consistent scoring system is not established to properly report an improvement of the surgical field compared with control groups.

### Duration of Surgery

Various reviews support that TXA greatly reduces operative time. This is not only clinically efficient but also reduces the patient's time under anesthesia, which may suggest shorter recovery periods. Moreover, a decrease in the DOS can help cut expenses for both the healthcare system and patients.^15^ This can be established by decreasing the amount of time needed to achieve hemostasis. In an SR, where TXA was used in aesthetic facial surgery, such as rhytidectomy, time to secure hemostasis was reduced by 25 to 60 min less.

On the contrary, an SR/MA reviewing TXA in patients undergoing closed and open rhinoplasty found no significant reduction of DOS after administrating TXA.^17^ This could be due to the fact that closed rhinoplasties usually require less time to perform than open rhinoplasty. Therefore, further subanalysis of these groups is warranted. Another SR/MA investigating the role of TXA on nasal surgeries showed similar results.^20^ One explanation may be that the DOS depends on the difficulty of the surgery and surgeon's skills.

### Postoperative Complications and Tranexamic Acid (TXA) Safety

TXA was found to reduce postoperative complications in rhinoplasty procedures in 7 reviews and in rhytidectomy in 5 reviews. However, it had no effect on reducing postoperative complications in patients who underwent blepharoplasty. In several reviews, the authors revealed that patients receiving TXA undergo less bruising and swelling, especially in the periorbital region, after rhinoplasty and rhytidectomy surgeries. This reduction in postoperative complications is advantageous for patient satisfaction, as it results in a faster healing time and improved appearance in the early postoperative period. Nonetheless, the authors of some published papers suggest that it is as effective as systemic corticosteroids like methylprednisolone or dexamethasone in reducing edema and ecchymosis. Nevertheless, it is not always superior.^19^

In addition, TXA in plastic surgery has a low incidence of adverse effects, which makes it safe for administration. TXA does not appear to pose a higher threat of thromboembolic events as is often associated with other antifibrinolytic drugs, which makes this agent a useful tool in managing intraoperative bleeding while not compromising patient safety. Based on the available literature, it has been demonstrated that the use of TXA is safe in patients who are undergoing plastic surgery and does not increase the risk of thromboembolic events.^3^ However, there are no papers that compare the safety of different antifibrinolytic agents to TXA in aesthetic surgeries. Furthermore, a study discussed the use of topical and local TXA in rhytidectomies, in order to prevent the occurrence of seizures and thromboembolic events. Surprisingly, they found that in patients who received local TXA mixed with tumescent solution and in patients where TXA was applied topically, they experienced skin necrosis, blistering, and delayed wound healing. After review of the literature, they found that topical TXA can affect cellular morphology and reduce cell adherence. They also concluded that wound healing complications are dose dependent, meaning the higher the TXA dose used, the less cellular metabolic activity observed. Another important point addressed is even though topical TXA is used in other facial aesthetic surgeries, there is no documented wound healing complications in procedures other than rhytidectomies. They hypothesize that this could be attributed to the lack of undermining in rhinoplasties and blepharoplasties. In addition, it could be because of skin thickness, as they observed that ischemia occurred in areas where the skin flap was thin and not in more medial areas where the flap was composite skin/superficial musculoaponeurotic system.^25^

### Gaps and Future Clinical Implications

After a comprehensive review of several SRs and MAs investigating the use of TXA in facial aesthetic surgery, it is evident that TXA is a very promising agent. However, there is a deficiency in the homogeneity of these papers. Some published papers include the use of TXA in nonaesthetic procedures, which is not the focus of this umbrella review. It is important to note that the 5 systematic reviews on blepharoplasty and TXA are all derived from a single study. Therefore, dedicated studies for the use of TXA in facial aesthetic surgery are recommended. In addition, to fully explore the potential of TXA on BLV, DOS, and postoperative complications, a standardized method of assessing these variables should be established. Measurement of BLV greatly varied in the papers reviewed. Some papers used blood-soaked gauze, while others used suction bottles or postoperative hemoglobin values as an assessment tool. Thus, having a unified tool for assessing and capturing the effect of TXA on facial aesthetic procedures should be established. In addition, the form, timing of administration, and dosage of TXA were not consistent in most of the studies. As a result, conclusions regarding the optimal TXA form and dose could not be obtained. Consequently, clear TXA administration guidelines should be explored and published. Another limitation is the extreme variability of the data collected; therefore, statistical analyses were not conducted. Finally, other agents should be explored to conclude whether TXA is the most suitable drug of choice in reduction of BLV, DOS, and postoperative complications of plastic surgery.

## CONCLUSIONS

To conclude, this umbrella review consolidates evidence that the use of TXA in facial aesthetic surgeries is indeed effective in reducing BLV and DOS. However, it is important to take into consideration that the included reviews varied in terms of assessing the BLV and DOS. Hence, this should be further taken into consideration to be unified in future studies to ensure valid information that will help enlighten surgeons in the future in assessing the utility of TXA in facial aesthetic surgeries.

## Supplemental Material

This article contains supplemental material located online at https://doi.org/10.1093/asjof/ojae105.

## Supplementary Material

ojae105_Supplementary_Data
