# Supplementary figures and images for: Tranexamic Acid Application in Facial Aesthetic Surgery: An Umbrella Review
Source: Aesthet Surg J Open Forum. 2024 Nov 7;6:ojae105. doi: 10.1093/asjof/ojae105 (PMC11630850; doi:10.1093/asjof/ojae105)

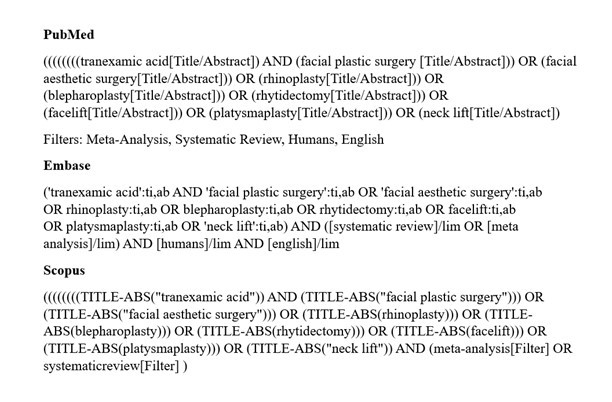

Supplement: ojae105_Supplementary_Data [file ojae105_supplementary_data.jpeg]
